# Adolescent menstrual cycle pattern, body mass index, endocrine and ovarian ultrasound characteristics of PCOS and future fertility, cardiovascular-, and metabolic health: a 25-year longitudinal follow-up study

**DOI:** 10.1093/humrep/deae262

**Published:** 2024-12-04

**Authors:** Machiel H A van Hooff, Mirte R Caanen, Henrike E Peters, Joop S E Laven, Cornelis B Lambalk

**Affiliations:** Department of Obstetrics and Gynaecology, Franciscus Gasthuis en Vlietland, Rotterdam, The Netherlands; Department of Reproductive Medicine, Amsterdam UMC, Vrije Universiteit, Amsterdam, the Netherlands; Department of Reproductive Medicine, Amsterdam UMC, Vrije Universiteit, Amsterdam, the Netherlands; Department of Obstetrics and Gynecology, Radboud University Medical Center, Nijmegen, the Netherlands; Division of Reproductive Endocrinology and Infertility, Department of Obstetrics and Gynaecology, Erasmus University Medical Center, Rotterdam, The Netherlands; Department of Reproductive Medicine, Amsterdam UMC, Vrije Universiteit, Amsterdam, the Netherlands

**Keywords:** adolescence, menstrual cycle irregularities, oligomenorrhea, prognosis, polycystic ovary syndrome, subfertility, metabolic syndrome, reproductive epidemiology

## Abstract

**STUDY QUESTION:**

What is the predictive value of oligomenorrhea and other PCOS diagnostic characteristics in adolescence (age 15–18 years) for future fertility and cardiovascular and metabolic health at adult age?

**SUMMARY ANSWER:**

Adolescents with oligomenorrhea are more often treated to conceive but are as likely to have as much children as those with regular periods, while persisting oligomenorrhea may associate more often with cardiovascular or metabolic problems.

**WHAT IS KNOWN ALREADY:**

Adolescents with oligomenorrhea have a high risk for adult PCOS associated with subfertility due to ovulatory disorders and long-term health risks. Longitudinal studies to estimate the extent of these risks with input starting at adolescence and covering the complete reproductive lifespan are lacking.

**STUDY DESIGN, SIZE, DURATION:**

A 25-year prospective follow-up study based on a unique population-based adolescent study on menstrual irregularities performed between 1990 and 1997, the Pubertal Onset of Menstrual Cycle abnormalities, a Prospective study (POMP study). Of the 271 invited adults, 160 (59%) participated.

**PARTICIPANTS/MATERIALS, SETTING, METHODS:**

We contacted stratified samples of the POMP study cohort two decades after the initial study for a questionnaire assessing PCOS features, fertility history, pregnancy outcome, metabolic, and cardiovascular health. One hundred and sixty subjects completed the questionnaire. The mean adolescent age was 15.3 years, and the women were 39.6 years at the time of follow-up. One hundred and eight subjects had a regular menstrual cycle as adolescents and 52 were oligomenorrheic.

**MAIN RESULTS AND THE ROLE OF CHANCE:**

Of those with adolescent regular menstrual cycles 12 never tried to conceive, 4 tried but never conceived and 92 of 96 (96%) conceived, 89 of 96 (93%) delivering at least one living child. The median number of children was two. The mean time to pregnancy (TTP) was 8.4 months in the women with regular periods as adolescents and 13.2 months in case of oligomenorrhea (*P* = 0.08) and subfertility was present in respectively 18% and 26%. 47 of 52 adolescents with oligomenorrhea tried to conceive and 45 succeeded to have at least one live birth. Twenty-eight per cent of the subjects reported a change over time of their menstruation pattern. Fifty per cent of the girls with adolescent oligomenorrhea developed a regular cycle and 16% of those with regular periods changed to oligomenorrhea with significantly more reported subfertility (40%, *P* = 0.04). In case of persistent oligomenorrhea, a significant proportion (40%) underwent fertility treatment (*P* = 0.04). Adult BMI did not differ between groups. The risk for pregnancy-induced hypertension or pre-eclampsia was comparable between the groups. Gestational diabetes developed in three subjects each with persistent oligo amenorrhea. Adult diabetes, hypertension, and hypercholesterolemia were also mostly reported in case of persistent oligomenorrhea. In this group, the prevalence of combined cardiovascular and metabolic problems was 14% compared to 7% in the case of regular menstrual cycles as adolescent.

**LIMITATIONS, REASONS FOR CAUTION:**

The numbers in the study are small. However, the small difference between the percentage with a least one living child of those with adolescent oligomenorrhea versus those with adolescent regular menstrual cycles is reassuring. Time to pregnancy data may have been biased by early treatment of oligomenorrheic adults.

**WIDER IMPLICATIONS OF THE FINDINGS:**

Oligomenorrheic adolescents may be reassured that their chance to have a live birth is comparable with those with a regular menstrual cycle.

**STUDY FUNDING/COMPETING INTEREST(S):**

This research received no external funding, J.S.E.L. received unrestricted research grants from the following companies (in alphabetical order): Ansh Labs, Ferring, Merck, and Roche Diagnostics. He received consultancy fees or royalties from Ansh Labs, Art pred, Ferring, Gedeon Richter, and Roche Diagnostics. He received presentation fees from Ferring and Roche Diagnostics as well as support for attending meetings and/or travel from Ferring and Roche Diagnostics and he participated in the advisory board of the LOCI Trial UK.

**TRIAL REGISTRATION NUMBER:**

Dutch Trial Registry, NTR5871.

## Introduction

Irregular menstrual cycles in adolescence are common and mainly a sign of physiological development from a premenarcheal state to regular ovulatory cycles (Southam and Richart, 1966; Treloar *et al.*, 1967; Vollman, 1977; van Hooff *et al.*, 2004). For a minority irregular menstrual cycles in adolescence are a symptom of endocrine disorders of the reproductive axis comparable with endocrine disorders in adults such as polycystic ovary syndrome (PCOS) (West *et al.*, 2014; Chan *et al.*, 2020; Caanen *et al.*, 2021). In general, long-term health consequences associated with diagnosed PCOS are subfertility, cardiovascular disease, and metabolic syndrome (Solomon, 1999; Wild *et al.*, 2000; Chavarro *et al.*, 2016; Meun *et al.*, 2020; Wekker *et al.*, 2020; Ollila *et al.*, 2023).

The association of adolescent irregular menstrual cycles with PCOS leads in early adulthood to uncertainty about future fertility. Early diagnosis of PCOS may provide an opportunity for early treatment and prevention of long-term health risks (Witchel *et al.*, 2015; Ibanez *et al.*, 2017). However, diagnosis of PCOS in puberty is difficult and a matter of ongoing debate (Caanen *et al.*, 2021). An incorrect diagnosis of a presumptive endocrine disorder may cause serious harm due to unfounded fears, a cause for psychological burden.

Aside from the difficulties of diagnosing PCOS at a young age, a pending question is to what extent individual features during adolescence associated with PCOS persist in the long term.

Data on subfertility and other long-term health problems in adults who experienced irregular menstrual cycles as adolescent are scarce and data concerning adolescent menstrual cycle pattern combined with adolescent endocrine and ovarian ultrasound data are lacking.

In this paper, we present results of the adult follow-up study of the Puberty Onset Menstrual cycle abnormalities, a Prospective study (POMP-study) (Caanen *et al.*, 2021) on the association of menstrual cycle pattern in early adolescence and subfertility, pregnancy outcomes, diabetes mellitus type II, and cardiovascular disease 25 years later.

The principal objective of the study was to estimate the predictive value of the adolescent menstrual cycle pattern alone and combined with clinical and/or biochemical signs of hyperandrogenism or polycystic ovarian morphology (PCOM) in adolescence (age 15–18 years) for the outcomes, infertility, subfertility, time to pregnancy, pregnancy outcome, metabolic, and cardiovascular health problems in adulthood.

## Materials and methods

We used the data obtained from the POMP prospective long-term adult follow-up study whose design was described in detail earlier (Caanen *et al.*, 2021). Here, we describe details relevant to this paper.

### Study population

The starting point was the data from a unique adolescent study executed between 1990 and 1997 (POMP) that collected data about menstrual cycle pattern, endocrinology, and body markers in adolescents in a general population. The material and methods are extensively described previously (van Hooff *et al.*, 1998, 1999, 2000a,b, 2004). The original cohort consisted of 2480 adolescent schoolgirls who were interviewed on their menstrual cycle pattern. A subset of the subjects was invited for physical examination, blood sampling for endocrine evaluation, and transabdominal ovarian ultrasound. All girls with oligomenorrhea were invited to participate in a follow-up visit after 3 years. For every girl with oligomenorrhea, two girls with regular menstrual cycles were invited as controls. The design of a case control study nested in a cohort study results in an efficient comparison between a cohort adolescents with oligomenorrhea (cases, low prevalence) and a cohort with regular menstrual cycles (controls, high prevalence). Adding more controls results only in a slightly higher statistical power despite much higher effort and costs.

For the long-term follow-up study, we identified the original participants (mean age, 15.1 years at entry of the study) who did not use hormonal contraceptives at the time of blood sampling (Caanen *et al.*, 2021). We approached these participants in 2016 to collect information regarding long-term follow-up. Data were obtained by an extensive postal questionnaire, and when information was not sufficient or consistent, by additional contact. In 160 of the 271 (59%) invited participants, we were able to obtain information about their history of fertility, type II diabetes, and cardiovascular disease. The responder group (n = 160) and non-responder group (n = 111) did not differ in adolescent baseline characteristics such as age, years after menarche, BMI, presence of hyperandrogenism, and oligomenorrhea (data not shown).

Both the POMP study and the long-term follow-up study (van Hooff *et al.*, 1998; Caanen *et al.*, 2021) were approved by the institutional review board, and informed consent was obtained from all subjects. The adult follow-up study was registered in the Dutch Trial Registry (trial registration number NTR5871).

### Adolescent parameters

Details on adolescent data collection were extensively described previously (van Hooff *et al.*, 1998, 2004). From the adolescent data, we categorized the menstrual cycle pattern in either oligomenorrhea (average menstrual cycle longer than 35 days) or regular cycles (average menstrual cycle shorter than 35 days). Gynaecological age was calculated by subtracting the age at menarche from the calendar age. All subjects were at least 2 years after menarche at the time they were classified as adolescent regular menstrual cycles or adolescent oligomenorrhea. Physical examination in adolescence consisted of measurement of height, weight, and waist-hip circumference. Hirsutism was defined as a modified Ferriman and Gallwey (mF&G) score of ≥8 (Ferriman and Gallwey, 1961); adolescent excess hair as mF&G ≥ 1 (Kiconco *et al.*, 2023). Acne was defined as a Plewig and Kligman score of ≥1 (Kligman and Plewig, 1976).

For endocrine evaluation, blood samples in adolescents with a regular menstrual cycle were taken between the first and the 10th day of the menstrual cycle. In oligomenorrheic girls, blood samples were also taken during the extended follicular phase but at least 21 days before the next period. Details about the assays used for endocrine evaluation were descried earlier (van Hooff *et al.*, 1999, 2000a; Caanen *et al.*, 2021).

Transabdominal ultrasound of the ovaries was performed in a subset of the adolescent study cohort, PCOM was defined as ≥ 10 antral follicles (2–8 mm) in one plane (Adams *et al.*, 1986; van Hooff *et al.*, 2000b) and/or a volume of ≥10 ml in at least one ovary, as was usual at the time of the investigation (Adams *et al.*, 1986).

### Adult follow-up study: the questionnaire

The questionnaire used in our follow-up study was an adaptation of a well-tested questionnaire used by the Department of Epidemiology of the Netherlands Cancer Institute in a Dutch cohort study on long-term effects of ovarian stimulation on IVF (de Boer *et al.*, 2005; van Leeuwen *et al.*, 2011) and reproductive outcomes in childhood cancer survivors (van den Berg *et al.*, 2018). The questionnaire addressed general health information, menstrual cycle characteristics, self-reported acne, and hirsutism (addressed by a mF&G score assessment).

The respondents who had a menstrual cycle longer than 35 days at the time of the questionnaire were considered to have oligomenorrhea (in case of hormonal contraception use, fertility treatment, pregnancy, or breastfeeding the menstrual cycle pattern during the year before these events was used). PCOS in adulthood was defined as oligomenorrhea and clinical hyperandrogenism (presence of hirsutism or severe acne) and/or reporting PCOS as medical diagnosis and/or reason for treatment by a physician (West *et al.*, 2014; Ollila *et al.*, 2019; Chan *et al.*, 2020; Caanen *et al.*, 2021).

Information concerning fertility was collected by questions about number of pregnancies, number of live births, time to pregnancy, treatment for subfertility or oligomenorrhea, and pregnancy outcomes. Subfertility was defined as a time to pregnancy of more than 12 months. Infertility was defined as the failure to conceive during the follow-up of the study. Questions regarding gynaecological health were about surgical treatment of uterus or ovaries, menopause, and current or earlier methods of contraception. Furthermore, information on medical history was collected specifically focused on medication, diabetes, hypertension, hypercholesterolemia, dyslipidemia, and cardiovascular disease. Cardiovascular disease was defined as a history of myocardial infarction, angina pectoris, cerebrovascular disease, or peripheral vascular disease.

### Statistical analysis

All statistical analyses were performed using SPSS version 29.0 (SPSS Inc., Chicago, IL, USA). Data on baseline characteristics, hormonal measurements, and ovarian morphology are reported as mean (SD) or median (interquartile range). Normal distribution was assessed visually by means of separate QQ plots for subgroups with regular menstrual cycles and oligomenorrhea.

**Figure 1.**
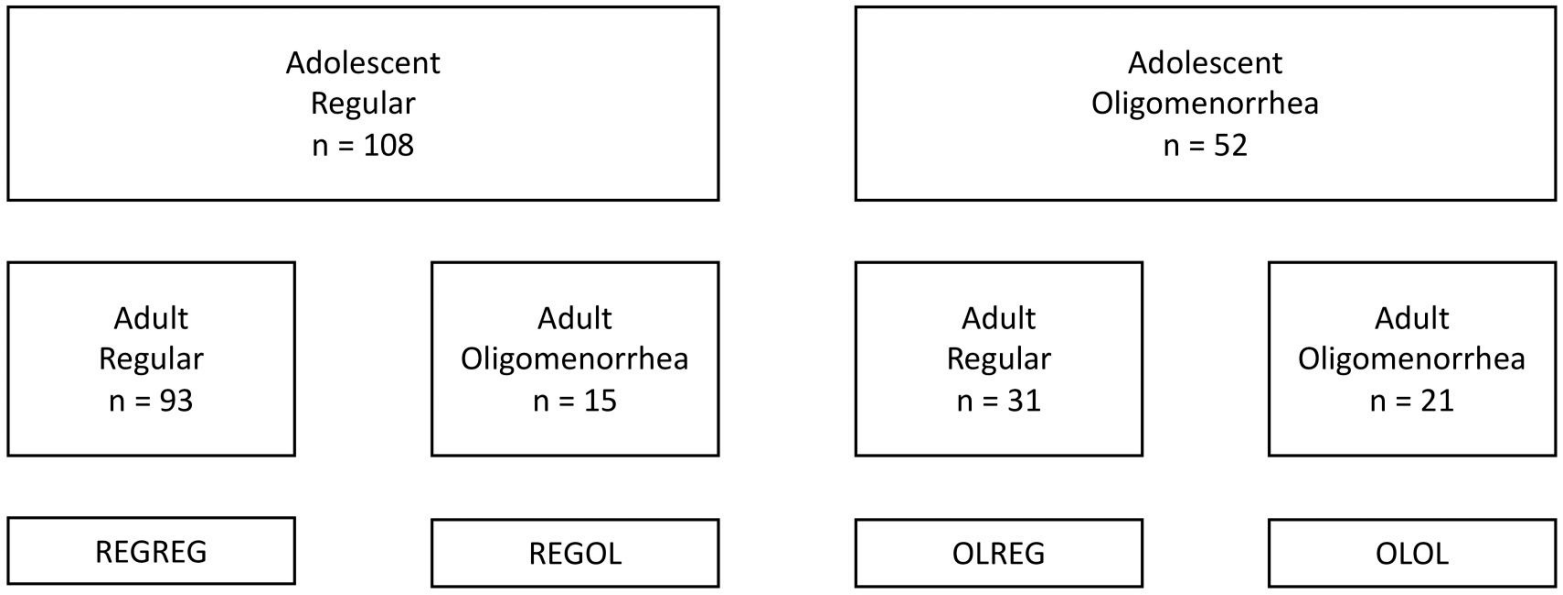
**Formation of subgroups based on adolescent and adult menstrual cycle pattern**.

We classified the subjects by menstrual cycle pattern in adolescence which resulted in those with adolescent oligomenorrhea and a group with a regular cycle at the time. However, it was from the beginning considered relevant to also study the impact of a change in the menstrual cycle classification from adolescence to adulthood over the period of follow-up. Therefore, we further classified subjects into subgroups based on a combination of their adolescent menstrual and adult menstrual cycle pattern: (i) those who had a regular menstrual cycle as adolescent and as an adult (REGREG), (ii) those who changed from a regular pattern to oligomenorrhea (REGOL), (iii) oligomenorrheic girls who developed a regular menstrual cycle pattern in adulthood (OLREG), and (iv) oligomenorrheic adolescents who remained oligomenorrheic (OLOL), Fig. 1.

To compare groups, we used one-way ANOVA for multiple subgroups or *t*-tests (normal distribution) and non-parametric Mann–Whitney tests when data were skewed. Chi-square analysis was used to compare sample frequencies.

Since adolescent oligomenorrhea is associated with subfertility, we performed binary logistic regression analysis with subfertility as the primary endpoint separately for both the adolescent oligomenorrhea group and the adolescent regular menstrual cycle group. Adolescent physical characteristics, endocrine, and ultrasound data were modelled as continuous variables or categorical data when appropriate.

Given previous reports (Zaadstra *et al.*, 1993; Rich-Edwards *et al.*, 1994) showing that the relation between adolescent BMI and adult subfertility is U-shaped, we performed an additional sub-analysis with adolescent BMI divided in four quartiles.

## Results

### Adolescent and adult characteristics

Adolescent and adult characteristics in groups based on adolescent menstrual cycle patterns are shown in Table 1. Anti-Mullerian hormone, LH, androstendione, prolactin, ovarian volume, and proportion of those with PCOM were higher and FSH lower at the time of adolescence in the oligomenorrheic group compared to those with a regular menstrual cycle (Table 1). As adults, we found no significant difference in clinical signs of hyperandrogenism such as adult acne or adult hirsutism. However, as we published previously, PCOS was diagnosed more often in those with adolescent oligomenorrhea (Caanen *et al.*, 2021). There were no differences in BMI or increase in BMI during the follow up. Possible confounders for subfertility such as smoking and alcohol intake were equal.

**Table 1.** Adolescent and adult characteristics according to menstrual cycle pattern in adolescence (age 15 years).

	*Adolescent menstrual cycle pattern*		
	Regular, n = 108	Oligomenorrhea n = 52	*P*-value
	*Adolescent characteristics*		
Age adolescent (years)	15.3 (0.6)	15.1 (0.5)	0.13
Age at menarche (years)	13.0 (1.0)	13.2 (1.0)	0.18
BMI (kg/m^2^)	20.3 (2.5)	20.0 (2.7)	0.44
Hip/waist ratio (cm/cm)	1.37 (0.08)	1.40 (0.08)	0.13
Acne*	67 (62%)	32 (62%)	0.96
Excess hair**	14 (13%)	11 (21%)	0.18
AMH (μg/l)	3.1 (2.2)	5.0 (2.9)	**<0.001**
LH (U/l)	3.2 (2.8)	5.2 (4.3)	**<0.001**
FSH (U/l)	5.2 (1.4)	4.7 (1.3)	**0.04**
Early follicular Estradiol (pmol/l)	140 (121)	156 (98)	0.43
Testosterone (nmol/l)	1.15 (0.5)	1.29 (0.44)	0.08
Androstendione (nmol/l)	5.2 (2.0)	6.0 (2.2)	**0.03**
DHEAS (μmol/l)	4.5 (2.4)	5.3 (2.0)	0.06
Prolactin (mU/l)	191 (92)	230 (111)	**0.02**
Insulin (mU/l)	9.9 (5.2)	11.2 (7.7)	0.30
Insulin/glucose ratio (ng/10^−4^ U)	2.1 (1.3)	2.7 (2.0)	0.08
Ovarian volume (cm^3^), n = 111	7.8 (2.8) n = 67	9.4 (4.6) n = 44	**0.04**
PCOM, n = 111	6 (9%)	21 (48%)	**<0.001**
	*Adult characteristics*		
Age adult (years)	39.8 (0.9)	39.3 (0.8)	**0.002**
BMI (kg/m^2)^	25.1 (4.5)	23.6 (4.4)	0.17
BMI increase	4.8(3.8)	4.0 (3.3)	0.23
Hirsutism (mF&G ≥ 8)***	6 (6%)	4 (8%)	0.60
Acne (self-reported)	18 (17%)	11 (21%)	0.49
PCOS	7(6%)	12(21%)	**0.004**
Smoking	8 (7%)	5(10%)	0.63
Alcohol	74(69%)	37 (71%)	0.73
High education level	69 (64%)	36 (69%)	0.51
Tubal or ovarian surgery	9 (8%)	4 (8%)	0.89

mF&G, modified Ferriman and Gallwey score; DHEAS, dehydroepiandrosterone; AMH, anti-Müllerian hormone; PCOM, polycystic ovary morphology; PCOS, polycystic ovary syndrome. Significant outcomes (*P* < 0.05) are presented in bold.

* Plewig and Kligman score ≥1.

** Modified Ferriman and Gallweyscore ≥ 1, addressed by researcher.

*** mF&G addressed through visual assessment by participant.

Data are presented as mean (SD) or number in subgroup (% in subgroup).

*P*-value for means: *t*-test; *P*-value for proportions chi-square test.

Ovarian or tubal surgery was performed in 8.1% (9/108) respectively 7.7% (4/52) of the adolescent regular menstrual cycle group and the adolescent oligomenorrhea group. None of the subjects had undergone laparoscopic endocoagulation of the ovaries as a treatment for anovulation.

### Adolescent menstrual cycle pattern and fertility outcomes, pregnancy complications, cardiovascular disease, and type 2 diabetes

**Figure 2.**
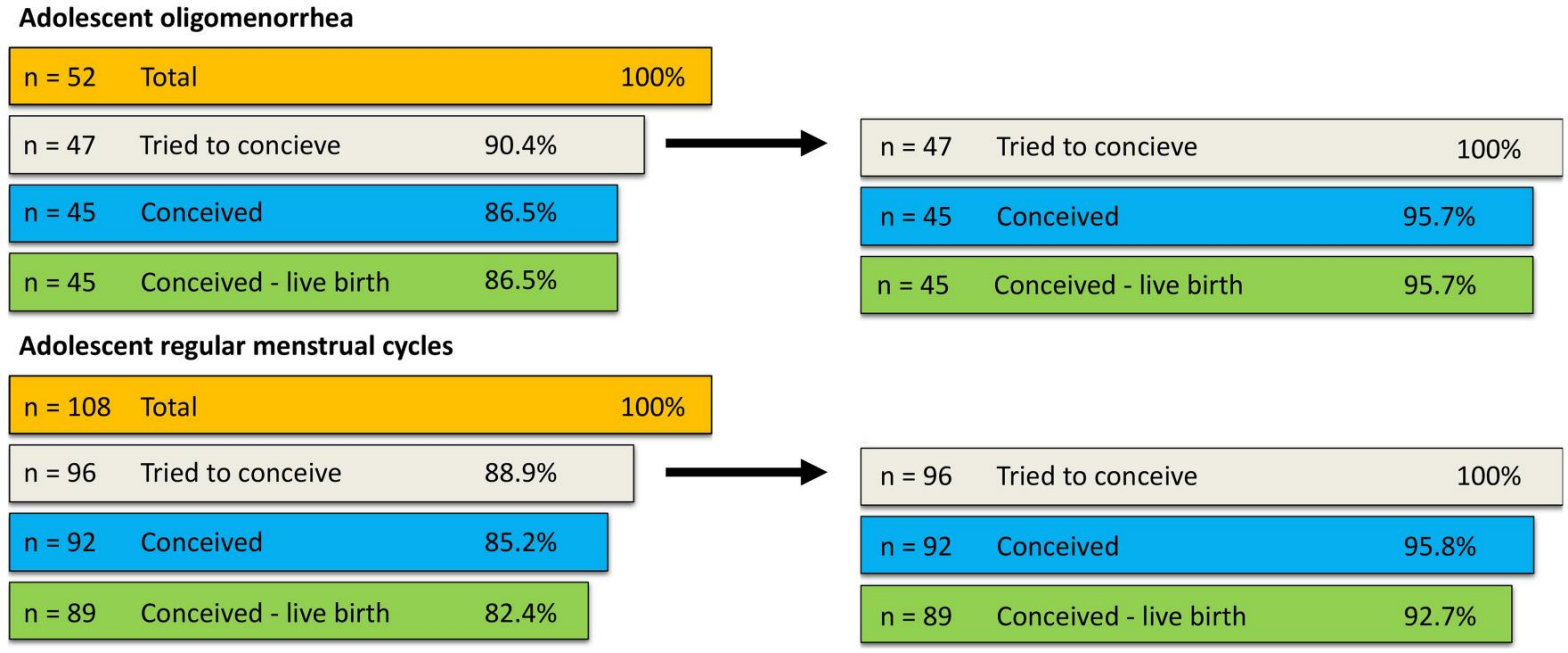
**Fertility outcomes.** Probability to have conceived and at least one live birth at age 38–40 years by menstrual cycle pattern in adolescence.

Of 108 adolescents with a regular menstrual cycle, 12 never tried to conceive and 4 tried but never conceived. Ninety-two who tried to conceive became pregnant (at least one pregnancy), of whom 89 gave birth to at least one living child (Fig. 2). Of subjects with adolescent oligomenorrhea five never tried to conceive; 47 tried to conceive and 45 (95.6%) of them succeeded to have at least one full-term pregnancy.

The prevalence of subfertility (defined as time to pregnancy longer than 12 months) did not differ significantly between those with regular menstrual cycles in adolescence (18%) or oligomenorrhea in adolescence (26%); odds ratio 1.6; 95% CI [0.7–3.7], Table 2. Those with oligomenorrhea in adolescence had fertility treatment twice as often compared to those with regular menstrual cycles in adolescence; 30% versus 15%; odds ratio 2.5; 95% CI [1.1–5.8], and six times more often fertility treatment related to ovulation disorders; 19% versus 3%; odds ratio 7.3; 95% CI [1.1–5.9]. Time to pregnancy was 5 months higher in the adolescent oligomenorrhea group (13.2 months) compared to the regular menstrual cycle group (8.4 months); *t*-test; *P* = 0.08, not significant. However, these data are skewed and the median time to pregnancy was 3 months for both adolescents with regular menstrual cycles and those with oligomenorrhea, Mann–Whitney *U*-test, *P* = 0.05. So, for those who did not become pregnant in the first 3 months, it took significantly longer to become pregnant for those with oligomenorrhea in adolescence than for those who had regular menstrual cycles.

**Table 2.** Fertility, pregnancy outcomes, and pregnancy complications, according to menstrual cycle pattern in adolescence (15 years).

	Adolescent menstrual cycle pattern		
	Regular	Oligomenorrhea	
	*Fertility and pregnancy outcomes*		
	n=96	n=47	***P*-value**
Conception	92 (96%)	45 (96%)	0.98
Conception, live birth	89 (93%)	45 (96%)	0.49
Subfertility >12 months	17 (18%)	12 (26%)	0.27
Fertility treatment (FT)	14 (15%)	14 (30%)	**0.03**
FT - related to ovulation disorder	3 (3%)	9 (19%)	**0.001**
Time to pregnancy—months mean (SD)*	8.4 (13.0)	13.2 (18.9)	0.08
Number of pregnancies (median)	2 (1-8)	3 (1-8)	0.18
Number full-term pregnancies (median)	2 (0-4)	2 (1-4)	0.21
Twin pregnancy	3(3%)	0	0.39
Stillborn neonate	1(1%)	1 (2%)	0.63
1 or more miscarriages	32 (33%)	15 (32%)	0.73
2 or more miscarriages	8 (9%)	6 (13%)	0.45
Abortion	6 (6%)	4 (9%)	0.67
Ectopic pregnancy	4 (4%)	1 (2%)	0.51
	*Pregnancy complications*		
	n=89	n=45	
Pregnancy induced hypertension including pre-eclampia	16 (18%)	8 (18%)	0.98
Gestational diabetes	0	3 (7%)	0.08
Caesarean section	19 (21%)	7 (16%)	0.43
	*Cardiovascular disease and type 2 diabetes*		
	n=108	n=52	
Diabetes type II	1 (1%)	1 (2%)	0.60
High cholesterol	3 (3%)	1(2%)	0.74
Hypertension	4 (4%)	4 (8%)	0.28
Coronary or vascular disease	1 (1%)	0	0.82
Combined	7 (7%)	5 (10%)	0.48

* Those with infertility (no conception) excluded.

Data are presented as mean (SD) or number in subgroup (% in subgroup).

*P*-value for means: *t*-test; *P*-value for proportions chi-square. Significant outcomes (*P* < 0.05) are presented in bold.

The frequency of hypertensive disorders in pregnancy or caesarean section did not differ between the groups. Gestational diabetes occurred only in three subjects who were all oligomenorrheic as adolescent.

In both groups, the composite prevalence of cardiovascular disease and type 2 diabetes was lower than 10% at the age of 38–40 years (Table 2).

### Fertility outcomes, pregnancy complications, and adult cardiovascular disease and diabetes type II according to menstrual cycle pattern in adolescence (15 years) and at age 38–40 years

Our results show that a significant number of 28% of the subjects had experienced a change in menstrual cycle pattern over time with implications for fertility performance outcomes as detailed in Table 3. Compared to those with a persistent regular menstrual cycle those that developed oligomenorrhea had a high rate of subfertility (40%) and a long time to pregnancy (17 months). Half of the women who had oligomenorrhea as adolescents developed a normal menstrual pattern but still experienced a prolonged mean time to pregnancy of 13 months comparable to women with persistent oligomenorrhea, 14 months. Fertility treatment, as often reported in relation to an ovulation disorder occurred mostly in case of persistent oligomenorrhea. Regarding pregnancy complications, there were except for three cases of gestational diabetes in the persistent oligomenorrhea group and none in any of the other groups no overt differences.

**Table 3.** Fertility outcomes, pregnancy complications, and adult cardiovascular disease and diabetes type II according to menstrual cycle pattern in adolescence (15 years) and at age 38–40 years.

	Adolescent menstrual cycle pattern				
	Regular		Oligomenorrhea		
	Adult menstrual cycle pattern				
	Regular	Oligomenorrhea	Regular	Oligomenorrhea	
Ado-adult subgroup	REGREG	REGOL	OLREG	OLOL	
	*Fertility outcome*				*P*-value
	n=81	n=15	n=26	n=21	
Subfertility >12 months	11 (14%)	6 (40.0%)	5 (19%)	7 (33%)	**0.04**
Fertility treatment (FT)	11 (14%)	3 (20%)	5 (19%)	9 (43%)	**0.03**
Ovulation disorder main reason for FT	3 (4%)	0 (0%)	2 (4%)	7 (33%)	**0.001**
Ovulation disorder mentioned.					
as main or secondary reason for FT	3 (4%)	2 (13%)	2(4%)	7(33%)	**0.003**
Time to pregnancy—months mean (SD)*	6.5 (9.6)	17.4 (22.1)	12.6 (19.6)	13.9 (18.4)	**0.05**
	*Pregnancy complications*				
	n=74	n=15	n=24	n=21	
Pregnancy induced hypertension including pre-eclampia	13 (18%)	3 (20%)	4 (15%)	4 (19%)	0.98
Gestational diabetes	0	0	0	3 (15%)	**0.001**
Caesarean section	16 (22%)	3 (20%)	3 (12%)	4 (19%)	0.77
	*Cardiovascular disease and type 2 diabetes*				
	n=93	n=15	n=31	n=21	
Diabetes type II	1 (1%)	0	0	1 (5%)	0.44
High Cholesterol	3 (3%)	0	0	1 (5%)	0.62
Hypertension	3 (3%)	1 (6%)	2 (7%)	2 (9%)	0.62
Coronary or vascular disease	1 (1%)	0	0	0	0.89
Combined	6 (7%)	1 (6%)	2 (7%)	3(14%)	0.66

* Those with infertility (no conception) excluded.

Data are presented as mean (SD) or number in subgroup (% in subgroup).

*P*-value for means: one-way ANOVA; *P*-value for proportions chi-square for multiple groups. Non-parametric Kruskal–Wallis test. Significant outcomes (*P* < 0.05) are presented in bold.

Long-term cardiovascular disease and DM II were not statistically significant but consequently more often reported in the group with persistent oligomenorrhea.

### Adolescent determinants to predict subfertility

Table 4 shows the results of the binary logistic regression analysis to predict subfertility for both adolescents with oligomenorrhea and regular menstrual cycles.

**Table 4.** Univariate and multivariate binary logistic regression to predict adult subfertility by adolescent menstrual cycle pattern classified at least 2 years after menarche.

Adolescent oligomenorrhea						
*Univariate analysi*s	B	S.E.	Wald	Odds Ratio	95% CI	*P*-value
Gynaecological age*	0.58	0.35	2.76	1.78	0.90–3.51	0.10
BMI	0.36	0.14	6.12	1.43	1.01–1.89	**0.01**
Waist-Hip ratio	3.51	4.42	0.63	33	0.00–189	0.43
mF&G score	−1.19	0.22	0.73	0.83	0.54–1.27	0.39
Acne	−1.55	0.763	4.10	0.21	0.05–0.95	**0.04**
LH	0.195	0.095	4.22	1.21	1.00–1.46	**0.04**
Testosterone	−2.69	0.892	0.91	0.76	0.13–4.39	0.76
Androstostenedione	0.35	0.117	3.82	1.41	1.00–2.00	**0.05**
DHEAS	−0.10	0.116	0.008	0.99	0.79–1.24	0.93
AMH	−0.003	0.68	0.001	1.003	0.79–1.27	0.98
Insulin/glucose ratio	−0.35	0.28	1.53	0.71	0.41–1.22	0.22
PCOM	0.86	0.28	1.21	2.36	0.51–10.9	0.27
*Multivariate analysis*						
BMI—*adjusted for Gynaecological age, Acne, LH, and Androstenedione*						
	0.32	0.16	3.86	1.38	1.00–1.89	**0.05**
BMI—*adjusted for Gynaecological age and smoking*						
	0.44	0.17	6.82	1.56	1.12–2.17	**0.009**

**Adolescent regular menstrual cycles**						

***Univariate analysis***	B	S.E.	Wald	Odds Ratio	95% CI	*P*-value

Testosterone	0.90	0.48	3.49	2.47	0.96–6.35	0.06
BMI	0.48	0.11	0.19	1.05	0.84–1.31	0.66

Other physical characteristics, endocrine, or ovarian ultrasound parameters did not contribute to improve the prediction for subfertility. Significant outcomes (*P* < 0.05) are presented in bold.

* Gynaecological age (age in months minus age in months at menarche).

mF&G, modified Ferriman and Gallwey score; DHEAS, dehydroepiandrosterone; AMH, anti-Mullerian hormone.

In univariate analysis entered as a continuous variable, higher BMI (*P* = 0.01), higher LH (*P* = 0.04), and higher androstenedione (*P* = 0.05) significantly contributed to predict adult subfertility in the adolescent oligomenorrhea subgroup. In univariate analysis, absence of acne (*P* = 0.04) also contributed significantly to predict adult subfertility in the adolescent oligomenorrhea subgroup which is counterintuitive as those with oligomenorrhea and no adolescent acne have a higher change for subfertility in this analysis.

In multivariate models with BMI entered as the first variable acne, LH and androstenedione had no additional significant contribution above BMI for predicting subfertility in the adolescent oligomenorrhea subgroup. Addition of possible confounders such as gynaecological age at the time of classification as adolescent oligomenorrhea and/or smoking to the multivariate models did not change this result.

In the adolescent regular menstrual cycle group, except for the testosterone level that nearly reached significance, *P* = 0.06, none of the parameters contributed significantly to the prediction of subfertility in adulthood. Adolescent BMI did not contribute to predicting subfertility in the adolescent regular menstrual cycle group.

### Adolescent menstrual cycle pattern, adolescent BMI, and adult subfertility

The BMI landmark for a higher chance for subfertility and the need for fertility treatment in the adolescent oligomenorrhea group lies around the 50th percentile (Table 5). Those with a BMI above the 50th percentile at age 15 years (19.6 kg/m^2^) have 40% risk for subfertility compared to 14% of those with a BMI below the 50th percentile, odds ratio 3.8 [0.96–15.4], (chi-square, 1 d.f.; *P* = 0.06). Forty-five per cent of oligomenorrheic adolescents with a BMI above the 50th percentile at age 15 years (19.6 kg/m^2^) needed fertility treatment compared to 18% of oligomenorrheic girls with a BMI below the 50th percentile; odds ratio 3.6 [0.98–13.3] (chi-square, 1 d.f.; *P* = 0.05).

**Table 5.** Impact of BMI in adolescence on the prediction of subfertility and need for fertility treatment in adulthood by adolescent menstrual cycle pattern.

	Adolescent BMI				
	<P25	P25–50	P50–75	>P75	
range BMI (kg/m^2^)	13.5–18.4	18.5–19.6	19.7–21.3	21.4–41.1	
	**Adolescent oligomenorrhea**				***P*-value**
Subfertility	n=14	n=13	n=8	n=12	0.67*
	2 (14%)	2 (15%)	3 (38%)	5 (42%)	
Fertility treatment	n=14	n=13	n=8	n=12	0.23**
	3 (21%)	2 (15%)	3 (38%)	6 (50%)	
	**Adolescent regular menstrual cycles**				
Subfertility	n=20	n=24	n=28	n=24	0.39
	5 (25%)	3 (13%)	3 (11%)	6 (25%)	
Fertility treatment	n=20	n=24	n=28	n=24	0.43
	2 (10%)	3 (13%)	3 (11%)	6 (25%)	

*P* (25, 25–50, 50–75, 75)=BMI percentile at age 15 years.

n=number in subgroup.

*P*-value chi-square test, 3 d.f.

* Adolescent oligomenorrhea subgroup: BMI >P50 at age 15 years (19.6 kg/m^2^) 40% subfertility compared to 14% with a BMI <P50 odds ratio 3.8 [0.96–15.4], (chi-square, 1 d.f.; *P* = 0.06).

** BMI >P50 at age 15 years (19.6 kg/m^2^) 45% with a needed fertility treatment compared to 18% with a BMI below the 50th percentile; odds ratio 3.6 [0.98–13.3] (chi-square, 1 d.f.; *P* = 0.05).

Those with regular menstrual cycles in adolescence with a BMI below the 25th percentile or above the 75th percentile, both have a chance of 25% of subfertility compared to a 12% chance for those with a BMI between the 25th and 75th percentile. These data fit to a U -shaped curve. The lower BMI subgroup did not need fertility treatment more frequently than those between the 25th and 75th percentile.

## Discussion

In this unique, 25 years, prospective, longitudinal cohort study, we provide data about the association of a number of features that classically also relate to PCOS such as oligomenorrhea, BMI, endocrine and ovarian ultrasound characteristics in early adolescence and fertility outcome, pregnancy outcomes, type II diabetes, and cardiovascular disease at the end of the fourth decade. It is the first longitudinal study that combines these data and covers the complete reproductive lifespan.

### Adolescent menstrual cycle pattern and fertility outcome

We found that as adults, adolescents with oligomenorrhea are just as likely to have children compared to adolescents with regular menstrual cycles. At the end of the fourth decade, adolescents with oligomenorrhea had the same number of children as those with at the time regular menstrual cycles that continued until the age of 40 years. In this context, it is of note that about half of the adolescent girls with oligomenorrhea developed a normal menstrual cycle. Furthermore, oligomenorrhea does not automatically implicate anovulation. Most long cycles in adolescents with irregular menstrual cycles are ovulatory (Peña *et al.*, 2018). Another reason why infertility may not be paramount is likely because being oligomenorrheic as adolescent may cause better fertility awareness leading to earlier and adequate treatment for example ovulation induction because of an ovulation disorder. Finally, some of the persistent oligomenorrheic participants will conceive spontaneously or after ovulation induction within 12 months before they are defined as subfertile.

To our knowledge, this is the first long-term follow-up study completely covering the fertile lifespan providing data about menstrual irregularity, especially oligomenorrhea, BMI, and PCOS features during adolescence and reproductive outcome.

Only one earlier study with a much shorter follow up reported on the relationship between adolescent menstrual irregularity (age 16 years) and fertility in early adulthood (age 26 years) (West *et al.*, 2014) and agrees with our findings showing no difference in childlessness, but a higher need for treatment for subfertility in those with adolescent menstrual cycle irregularities.

### Role of adolescent BMI, adolescent menstrual cycle disorders, and fertility outcome

Adolescent BMI is the most frequently studied univariate determinant to predict subfertility, oligomenorrhea, or PCOS in adulthood (Zaadstra *et al.*, 1993; Rich-Edwards *et al.*, 1994; Gesink Law *et al.*, 2007; Polotsky *et al.*, 2010; Chavarro *et al.*, 2016).

Our data confirm the important role of a higher adolescent BMI in the prediction of subfertility (Jokela *et al.*, 2007; Laru *et al.*, 2021). With logistic regression analysis, adolescent BMI significantly contributed to predict subfertility in oligomenorrheic adolescents.

Several studies indicate reduced fertility in those with a BMI lower or higher than normal during adolescence(Rich-Edwards *et al.*, 1994; Laru *et al.*, 2021). Without reaching statistical significance though we found a similar U-shaped effect for the relation between adolescent BMI and future fertility for those with adolescent regular menstrual cycles, but not for those with oligomenorrhea in adolescence.

The relationship between adolescent oligomenorrhea, adolescent BMI, and adult subfertility is complex. Until now, no previous study provided combined data about both adolescent BMI and adolescent menstrual cycle pattern to predict adult subfertility. It seems that especially those with adolescent oligomenorrhea in the upper half of the adolescent BMI distribution (BMI>P50; ≥ 19.6 kg/m^2^) had a high (40%) chance for subfertility indicating that also in that regard both are indeed apparently substantially related even when no real overweight or obesity is involved. Although the adolescents in our study had a higher BMI than peers, the majority were not obese or even overweight and comparable with other recently published European cohorts (Ollila *et al.*, 2016; Laru *et al.*, 2021). Australian adolescents of 15 years in the RAINE cohort showed a mean BMI of 22.7 kg/m^2^ (Hickey *et al.*, 2011). They found a significantly higher adolescent BMI in adolescents with PCOS using the Rotterdam criteria (24.5 kg/m^2^ (5.7)) or the Androgen Excess Society criteria (28.8 kg/m^2^ (6.7)) compared to controls (22.3 kg/m^2^ (3.0)).

In our study, the mean gain in weight and BMI was not higher for those with adult oligomenorrhea compared with those with adult regular menstrual cycles. By contrast, an increased gain in BMI between age 14 and 31 years for those with self-reported oligomenorrhea, hyperandrogenism, and PCOS compared to referents is reported by Ollila *et al.* (2016).

### Endocrine and ovarian ultrasound characteristics of PCOS in early adolescence and fertility outcome

In the univariate logistic regression analysis, we found that adolescent testosterone level may predict subfertility in the adolescent regular menstrual group, but not in the oligomenorrhea group. Without accounting for adolescent menstrual cycle patterns, it was previously shown that higher total testosterone concentrations in the first postmenarcheal years were associated with lower fertility in the third decade (Apter and Vihko, 1990). On the other hand, others found no association between testosterone concentration at age 16 years and fertility problems at age 26 years (West *et al.*, 2014).

So, high androgens may predict adult subfertility in adolescents with regular menstrual cycles and in the general population without accounting for menstrual cycle pattern but not for those with known adolescent oligomenorrhea. We speculate that this have been the consequence of the higher pre-test risk for subfertility in oligomenorrheic adolescents which makes it more difficult to further improve this prediction by other parameters. With regard to the interpretation of the predictive value of testosterone, limitations of our study and the study by Apter are the immunoassay way it was measured. Both studies reported total testosterone. West *et al.* also reported total testosterone but measured with Agilent triple quadrupole 6410 LC/MS equipment with an electrospray ionization source operating in positive-ion mode (Agilent Technologies, Wilmington, DE, USA). Nowadays reporting free testosterone measured by LC-MS is considered as standard (Peña *et al.*, 2020).

The predictive value of absence of acne in the adolescent oligomenorrhea group is counterintuitive. Acne was scored as yes or no. No classification of the severity of acne was available. Mild comedonal acne is very common among adolescents. Only moderate to severe comedonal acne or inflammatory acne is likely to relate to hyperandrogenism (Slayden *et al.*, 2001) and could not be analysed separately from our data.

Especially adolescent oligomenorrhea, BMI, and testosterone play a role in the prediction of subfertility. This is remarkably in line with the international evidence-based guideline for the assessment and management of PCOS that recommends adolescent oligomenorrhea and testosterone to diagnose adolescent PCOS (Peña *et al.*, 2020; Teede *et al.*, 2023).

### Adolescent menstrual cycle disorders and adult metabolic or cardiovascular health

By the end of the fourth decade, we found no elevated prevalence of cardiovascular or metabolic disease among those with adolescent oligomenorrhea. Several earlier studies have demonstrated significant associations between menstrual irregularity in the early twenties and the development of cardiovascular disease or type 2 diabetes in the following decades (Solomon *et al.*, 2002; Glueck *et al.*, 2015; Wang *et al.*, 2020; Wekker *et al.*, 2020; Wang *et al.*, 2022). Pinola *et al.* (2012) found that in a 16-year-old population menstrual disorders are a good marker for hyperandrogenaemia, and that higher androgen levels at this young age are associated with an adverse lipid profile early in life.

Most likely, the distribution of causes for oligomenorrhea in our young age group is different from the distribution in studies with young adult women in whom the cause of the abnormal menstrual cycle pattern (e.g. PCOS or early menopause) is more likely associated with cardiovascular disease (Wang *et al.*, 2020). Of note though, in our subgroup with persistent oligomenorrhea the prevalence of metabolic or cardiovascular disease was twice as high at the age of 40 years compared to the other subgroups, but not statistically significant.

### Limitations and strengths

An overt limitation of our research is the low number of subjects in the subgroups which resulted in wide confidence intervals, hindered multivariate analyses, and may not have led to the identification of well-known associations as significant. Another limitation is that all adult data were collected by questionnaires and no adult physical examination, endocrine- and metabolic evaluation, or ultrasound investigation was performed, which would have provided more reliable data. Given the design of the study, data are only applicable for Caucasian especially Northwest European women. In the early nineties, 90% of the adolescent cohort belonged to this ethnic group and the analysis was limited to this population. Clearly, the end of the fourth decade is too early to draw conclusions about adolescent menstrual cycle irregularity and risk for premature or more severe metabolic or cardiovascular disease in adulthood. For this association our data should be interpreted with caution and valuated as exploratory only.

Finally, although all the features studied here are classically part of PCOS, due to limited numbers our study did not allow meaningfully linking adolescent PCOS diagnosis perse to lifetime fertility outcome.

Important strengths of the study are the population-based and longitudinal design with 25 years follow-up, the diligent collection of data during adolescence and the amount of data per individual including endocrine and ultrasound data at the adolescent age. This offers the unique possibility to weigh the importance of various parameters in not earlier presented multivariate models.

## Conclusions

Oligomenorrheic adolescents may be reassured that their chance to have a live birth is not different compared to those with regular periods. Oligomenorrheic adolescents have a higher than 50% chance for natural conception and when they are subfertile, treatment is highly successful. Those with a higher BMI than peers will have a longer time to pregnancy and a higher chance to need treatment for ovulation disorders.

In adolescents with regular menstrual cycles, high testosterone levels may predict subfertility. In general, testosterone levels will only be available for those who seek medical care for menstrual cycle disorders, hirsutism, or severe acne or in scientific research. Its role in the prediction of subfertility on a population level is limited.

We emphasize the importance of population-based studies as compared to hospital-based studies. They provide additional data about girls and women who will not visit their physician or gynaecologist such as adolescents with regular menstrual cycles and oligomenorrheic women who have a short time to pregnancy.

## Data Availability

The datasets generated during and/or analysed during the current study are not publicly available but are available from the corresponding author on reasonable request.
